# Characterizing healthcare utilization patterns in a Danish population with headache: results from the nationwide headache in Denmark (HINDER) panel

**DOI:** 10.1186/s10194-023-01553-w

**Published:** 2023-02-24

**Authors:** Thien Phu Do, Mikala Dømgaard, Simon Stefansen, Timothy J. Steiner, Messoud Ashina

**Affiliations:** 1 Department of Neurology, Danish Headache Center, Copenhagen University Hospital - Rigshospitalet, Copenhagen, Denmark; 2 Danish Knowledge Center On Headache Disorders, Glostrup, Denmark; 3 Department of Clinical Medicine, University of Copenhagen, Copenhagen, Denmark; 4 Department of Neuromedicine and Movement Science, Norwegian University of Science and Technology, Trondheim, Norway; 5 Division of Brain Sciences, Imperial College London, London, UK

**Keywords:** Headache disorders, Disease burden, Healthcare utilization, Barriers to care, Population survey, Denmark

## Abstract

**Introduction:**

Worldwide, far from all of those who would benefit make use of headache services, largely because of clinical, social, and political barriers to access. Identifying the factors contributing to low healthcare utilization can generate evidence to guide health policy. Our purpose here is better to characterize healthcare utilization patterns in Denmark.

**Methods:**

The Headache in Denmark (HINDER) study is a nationwide cross-sectional survey of people with headache, conducted using SurveyXact (Rambøll Group A/S, Copenhagen). Healthcare utilization was assessed in a study sample generated by population screening and recruitment. Data collection occurred over two weeks, from September 23^rd^ until October 4^th^, 2021. The questions enquired into disease characteristics, management, burden, medication intake and healthcare utilization.

**Results:**

The number of participants included in the HINDER panel was 4,431, with 2,990 (67.5%: 2,522 [84.3%] female, 468 [15.7%] male; mean age 40.9 ± 11.6 years) completing the survey. One quarter of participants (27.7%) disagreed or strongly disagreed that they were able to manage their headache attacks. Most participants (81.7%) agreed or strongly agreed that their headache was a burden in their everyday lives. The most reported acute medications, by 87.2% of participants, were simple analgesics; of note, 8.6% reported using opioids for their headache. One quarter of participants (24.4%) had never consulted a medical doctor for their headache; one in six (16.5%: more than two thirds of the 24.4%) had never done so despite agreeing or strongly agreeing that their headache was a burden in their everyday lives. Two thirds (65.3%) of participants overall, and almost three quarters (72.4%) of those with weekly headache, had tried one or more complementary or alternative therapies outside conventional medical care.

**Conclusions:**

Our findings are indicative of inadequate delivery of headache care in a country that provides free and universal coverage for all its residents. The implications are twofold. First, it is not sufficient merely to make services available: public education and increased awareness are necessary to encourage uptake by those who would benefit. Second, educational interventions in both pre- and postgraduate settings are necessary, but a prerequisite for these is a resetting of policy priorities, properly to reflect the very high population ill-health burden of headache.

## Introduction

Headache disorders directly affect more than 1 billion people across the world and constitute a leading cause of disability [1–4]. This negative impact is largely avoidable, with a range of effective and cost-effective treatments available, even in low-to middle-income countries (LMICs). Worldwide, however, far from all of those who would benefit make use of these therapies, largely because of clinical, social and political barriers to access [3–5].

Many of those with tension-type headache or migraine, the most prevalent headache disorders, can adequately self-manage, if provided with a little knowledge and access to the appropriate over-the-counter medications [6–8]. In countries where headache services exist at all, their focus is usually on specialist (tertiary) care. This is clinically and economically inappropriate: most headache disorders can effectively and more efficiently (and at lower cost) be treated in educationally supported primary care, with only complicated cases requiring referral to specialist care [3, 4, 9]. At the same time, compartmentalizing divisions between primary, secondary and tertiary care in many healthcare systems create multiple inefficiencies, confronting patients attempting to navigate these levels (the “patient journey”) with perplexing obstacles. Seeking consultation in primary care is therefore the first essential step in the patient pathway to care. Despite the barriers referred to [3, 5], headache is the most common presenting neurological symptom in primary care [3].

In Denmark, a country with well organized, highly resourced, and readily accessible services [10], a nationwide survey identified a low rate of healthcare utilization, even among people with headache occurring once or more every week, in parallel with both a high rate of lost productivity at work and lack of understanding from those in their close environment (family, friends, work colleagues and employers) [11].

Our purpose here is better to characterize healthcare utilization patterns in Denmark by conducting a nationwide cross-sectional survey of people with headache. Identifying the factors contributing to low healthcare utilization can generate evidence to guide health policy. Potential benefits of rational health policies are not limited to the affected individual: they also promote better use of resources since headache disorders are a primary driver of losses to economies [3]. Indeed, evidence suggests that delivery of structured headache care is highly cost-effective, and may be cost-saving [3].

## Methods

### Overview

The Headache in Denmark (HINDER) study is a nationwide cross-sectional survey of people with headache, conducted using SurveyXact (Rambøll Group A/S, Copenhagen). Healthcare utilization was assessed in a study sample generated by population screening and recruitment. Data collection occurred over two weeks, from September 23^rd^ until October 4^th^, 2021.

### Ethics

Conduct of surveys is exempt from processing by the National Committee on Health Research Ethics in Denmark. Survey data were handled confidentially, and anonymity of respondents was maintained throughout the study.

### Screening and recruitment

The screening and recruitment phase commenced on May 3^rd^, 2021, and closed on June 30^th^, 2021. We used social media (Facebook) to publicize and drive a recruitment campaign, with no predetermined prioritization of age or gender. Users of Facebook in Denmark aged ≥ 18 years were therefore the sampled population. Of 4.8 million people in Denmark meeting the age criterion, approximately 3.3 million were estimated to have a Facebook account [12]. Moreover, participants in a previous survey also recruited through Facebook were invited [11].

On accessing the invitation, users were asked to complete a Screening Module, which was a brief questionnaire capturing headache and demographic information (headache frequency, age and gender, with a median completion time of 128 s). Eligible participants (those meeting the age criterion and reporting at least one headache day in the prior year) were then invited to participate and become members of the HINDER panel, which was active until the end of 2022.

### Questionnaire and enquiry

The HINDER study module on healthcare utilization patterns was developed by the Danish Knowledge Center on Headache Disorders in collaboration with clinicians and experts in headache disorders from the Danish Headache Center. The questions in the Healthcare Utilization Module (Table 1), with a target audience of people with headache, enquired into disease characteristics, management, burden, medication intake and healthcare utilization. Headache diagnosis was self-reported.

**Table 1:** The healthcare utilization module

**Headache type**	
What type of headache do you have? (You may select more than one option) [one or more]	• I do not know what specific type of headache I have • Tension-type headache • Migraine • Horton's headache (cluster headache) • Post-traumatic headache (after head or neck injury) • Other type of headache
**Disease duration**	
How long have you lived with headache? [one only]	• Less than 1 year • 1–5 years • More than 5 years
**Headache frequency**	
How often do you usually have headache? [one only]	• At least once a week • A couple of times a month • A couple of times a year • Less frequently
**Management**	
I am able to manage my headache attacks well [one only]	• Strongly agree • Agree • Neither agree nor disagree • Disagree • Strongly disagree
**Burden**	
My headache is a burden on my everyday life [one only]	• Strongly agree • Agree • Neither agree nor disagree • Disagree • Strongly disagree
**Acute medication intake**	
What medication do you take when you have a headache? (You may select more than one option) [one or more]	• Migraine medications (e.g., sumatriptan, eletriptan, relpax, rizatriptan, maxalt, zolmitriptan or other triptan) [if respondent reported migraine] • Over-the-counter/simple analgesics (e.g., ibuprofen, ipren, paracetamol, pinex, pamol, naproxen, combination medications) • Strong analgesics (e.g. codeine, tramadol, oxycodone, morphine) • Other • I do not take pain medications [Brand names were listed in addition to generic names]
**Healthcare utilization (conventional medical care)**	
How long did it take from the time of your onset of headache until you consulted your doctor? [one only]	• I have not been to the doctor for my headache • Up 1 year • Up to 5 years • More than 5 years
**Healthcare utilization (complementary and alternative medicine)**	
Have you sought a different type of treatment provider for your headache than your general practitioner/other medical doctor? [one only]	• No, never • Yes, one other provider • Yes, several different providers

The questionnaire employed branching logic to generate additional specific questions (Table 2). These included questions on knowledge of triptans, reason(s) for not having consulted primary care for headache, and, among those who did consult, level of care where headache was managed, frequency of contacts with headache care services, and perceived quality of care.

**Table 2:** Healthcare utilization module (branching logic questions)

**If participant reported migraine, but no usage of triptans**	
Triptans are a type of medication you can use for acute migraine attacks. Have you heard of triptans before? (e.g., sumatriptan, eletriptan, relpax, rizatriptan, maxalt, zolmitriptan or other) [one only]	• Yes, I have tried them before, but I am not using them any longer • Yes, but I have never tried them • No, I have no knowledge about them
**If participant reported ‘I have not been to the doctor for my headache**	
You have indicated that you have not seen a doctor for your headache. What is the reason? [one only]	• My headache is not severe enough to go to the doctor • I have considered talking to my doctor but have not come around to doing it • I do not know if my doctor can help me with my headache • I do not need my doctor's assistance to manage my headache • Other
**If participant reported that they had sought medical advice for their headache**	
Where is your headache treatment/follow-up currently taking place primarily? [one only]	• At my general practitioner • At a neurologist • At a neurological department/ headache clinic
When was the last time you went to a medical doctor for your headache? [one only]	• Less than 1 year • 1–3 years • More than 3 years ago
After consulting the medical doctor for my headache, I can better handle my headache attacks [one only]	• Strongly agree • Agree • Neither agree nor disagree • Disagree • Strongly disagree
After consulting the medical doctor for my headache, I have more knowledge about my headache [one only]	• Strongly agree • Agree • Neither agree nor disagree • Disagree • Strongly disagree

We excluded participants who did not complete all questions.

### Statistical methods

We used descriptive statistics to compare healthcare utilization patterns according to survey demographics and reported headache frequency (occurring once or more per week, month or year). Continuous and count outcomes are presented using means with standard deviations. Binary and multinomial outcomes are presented with absolute numbers and percentages. All analyses were conducted calculated using IBM SPSS Statistics, version 28.0.0.0 (190).

## Results

### Study population and participating proportion

**Fig. 1:**
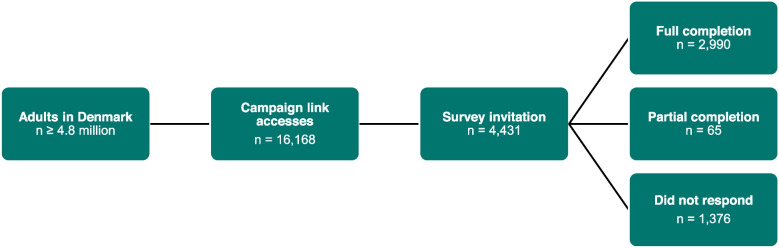
Participant flow diagram. The campaign link was accessed 16,168 times with 4,708 acceptances of the invitation to participate. After exclusion of duplicates, the number of participants included in the HINDER panel was 4,431, of whom 2,990 completed all questions of the Healthcare Utilization Module

Out of a population of approximately 4.8 million adults in Denmark, the campaign link to the HINDER panel was accessed 16,168 times, with 4,708 acceptances of the preliminary invitation. After exclusion of duplicates, the number included in the HINDER panel was 4,431. All received the invitation to participate in the Healthcare Utilization Module, with 2,990 (67.5%: 2,522 [84.3%] female, 468 [15.7%] male; mean age 40.9 ± 11.6 years) completing all questions (Fig. 1; Table 3).

**Table 3:** The healthcare utilization module

Enquiry	Response according to reported headache frequency				
**How often do you have headache (past year)?**	**All participants** *n* = 2,990 (100%)	**At least once a week** *n* = 1,881 (62.9%)	**A couple of times a month** *n* = 972 (32.5%)	**A couple of times a year** *n* = 129 (4.3%)	**Less frequently** *n* = 8 (0.3%)
**Age, years**					
Mean ± SD	40.9 ± 11.6	40.2 ± 11.7	41.8 ± 11.1	45.3 ± 12.6	48.1 ± 11.2
**Proportion, n (%)**					
**Gender**					
Male	468 (15.7%)	280 (14.9%)	147 (15.1%)	37 (28.7%)	4 (50.0%)
Female	2,522 (84.3%)	1,601 (85.1%)	825 (84.9%)	92 (71.3%)	4 (50.0%)
**Headache type**					
Tension-type headache	1,825 (61.0%)	1,218 (64.8%)	563 (57.9%)	43 (33.3%)	1 (12.5%)
Migraine	1,714 (57.3%)	1,046 (55.6%)	588 (60.5%)	78 (60.5%)	2 (25.0%)
Cluster headache	103 (3.4%)	69 (3.7%)	25 (2.6%)	7 (5.4%)	2 (25.0%)
Post-traumatic headache	316 (10.6%)	281 (14.9%)	35 (3.6%)	0 (0.0%)	0 (0%)
Other headache disorder	271 (9.1%)	191 (10.2%)	69 (7.1%)	11 (8.5%)	0 (0%)
Unknown	477 (16.0%)	295 (15.7%)	154 (15.8%)	25 (19.4%)	3 (37.5%)
**I am able to manage my headache attacks well**					
Strongly agree	116 (3.9%)	44 (2.3%)	51 (5.2%)	18 (14.0%)	3 (37.5%)
Agree	961 (32.1%)	488 (25.9%)	414 (42.6%)	55 (42.6%)	4 (50.0%)
Neither agree nor disagree	1,084 (36.3%)	727 (38.6%)	319 (32.8%)	38 (29.5%)	0 (0%)
Disagree	699 (23.4%)	513 (27.3%)	171 (17.6%)	14 (10.9%)	1 (12.5%)
Strongly disagree	130 (4.3%)	109 (5.8%)	17 (1.7%)	4 (3.1%)	0 (0%)
**My headache is a burden on my everyday life**					
Strongly agree	1061 (35.5%)	836 (44.4%)	208 (21.4%)	16 (12.4%)	1 (12.5%)
Agree	1381 (46.2%)	857 (45.6%)	490 (50.4%)	33 (25.6%)	1 (12.5%)
Neither agree nor disagree	352 (11.8%)	138 (7.3%)	178 (18.3%)	36 (27.9%)	0 (0%)
Disagree	156 (5.2%)	39 (2.1%)	85 (8.7%)	31 (24.0%)	1 (12.5%)
Strongly disagree	40 (1.3%)	11 (0.6%)	11 (1.1%)	13 (10.1%)	5 (62.5%)
**Acute medication intake**					
Triptans	991 (33.1%)	648 (34.4%)	302 (31.1%)	39 (30.2%)	2 (25.0%)
Simple analgesics	2,608 (87.2%)	1,642 (87.3%)	866 (89.1%)	96 (74.4%)	4 (50.0%)
Strong analgesics (opioids)	256 (8.6%)	190 (10.1%)	59 (6.1%)	6 (4.7%)	1 (12.5%)
Other	240 (8.0%)	167 (8.9%)	58 (6.0%)	13 (10.1%)	2 (25.0%)
Do not use	731 (24.4%)	107 (5.7%)	30 (3.1%)	9 (7.0%)	3 (37.5%)
**Health utilization (primary care)**^**1**^					
Never	731 (24.4%)	369 (19.6%)	313 (32.2%)	44 (34.1%)	5 (62.5%)
< 1 year	1,121 (37.5%)	802 (42.6%)	283 (29.1%)	35 (27.1%)	1 (12.5%)
< 5 years	689 (23.0%)	418 (22.2%)	242 (24.9%)	29 (22.5%)	0 (0%)
> 5 years	449 (15.0%)	292 (15.5%)	134 (13.8%)	21 (16.3%)	2 (25.0%)
**Health utilization (complementary and alternative medicine)**^**2**^					
Never	1,037 (34.7%)	518 (27.5%)	433 (44.5%)	79 (61.2%)	7 (87.5%)
One treatment/provider	792 (26.5%)	478 (25.4%)	285 (29.3%)	28 (21.7%)	1 (12.5%)
Several treatments/providers	1,161 (38.8%)	885 (47.0%)	254 (26.1%)	22 (17.1%)	0 (0%)

^1^How long did it take from the time of your debut of headache until you consulted your primary care physician/general practitioner?

^2^Have you sought a different type of treatment/provider for your headache than your general practitioner/other medical doctor?

### Headache disorders

The most commonly reported headache disorders were tension-type headache (*n* = 1,825 [61.0%]) and migraine (*n* = 1,714 [57.3%]) (Table 3), followed by post-traumatic headache (*n* = 316 [10.6%]) and cluster headache (*n* = 103 [3.4%]). “Other headache disorder” (unspecified) was reported by 271 (9.1%), while a further 477 (16.0%) participants did not know which headache disorder they had.

### Management

One quarter of participants (27.7%) disagreed or strongly disagreed that they were able to manage their headache attacks (Table 3). This was clearly related to headache frequency: those with highest frequency were most likely to report disagreement (Table 3).

### Burden

Most participants (81.7%) agreed or strongly agreed that their headache was a burden in their everyday lives (Table 3). Those with less frequent headache were less likely to agree (Table 3).

### Acute medications

The most-reported acute medications, by 87.2% of participants, were simple analgesics (Table 3). Of note, 8.6% reported using opioids for their headache, with the highest proportion (10.1%) found among those with weekly headache.

Of participants reporting migraine, 56.9% also reported using triptans for their attacks (Table 4). Of the 43.1% who did not, two thirds (68.3%) had never tried triptans and almost half (44.1%) had never heard of triptans before.

**Table 4:** Triptan usage in respondents with migraine

Enquiry	Response according to reported headache frequency				
**How often do you have headache (past year)?**	**All participants** *n* = 1,714 (100%)	**At least once a week** *n* = 1,046 (61.0%)	**A couple of times a month** *n* = 588 (34.4%)	**A couple of times a year** *n* = 78 (4.6%)	**Less frequently** *n* = 2 (0.1%)
**Age, years**					
Mean ± SD	40.3 ± 11.0	39.5 ± 11.1	41.1 ± 10.6	45.1 ± 11.4	46 ± 1.4
**Proportion, n (%)**					
**Gender**					
Male	176 (10.3%)	102 (9.8%)	56 (9.5%)	17 (21.8%)	1 (50%)
Female	1538 (89.7%)	944 (90.2%)	532 (90.5%)	61 (78.2%)	1 (50%)
**Triptan usage**					
Yes	975 (56.9%)	633 (60.5%)	301 (51.2%)	39 (50%)	2 (100%)
No	739 (43.1%)	413 (39.5%)	287 (48.8%)	39 (50%)	0 (0%)
**Participants with migraine and no triptan usage, *n* = 739**					
Have tried triptans, but do not use them anymore	234 (31.7%)	138 (33.4%)	89 (31.0%)	7 (17.9%)	N/A
Have heard of triptans, but never tried	179 (24.2%)	107 (25.9%)	62 (21.6%)	10 (25.6%)	N/A
Never heard of triptans	326 (44.1%)	168 (40.7%)	136 (47.4%)	22 (56.3%)	N/A

### Healthcare utilization (conventional medical care)

One quarter of participants (24.4%) had never consulted a medical doctor for their headache (Table 3). Even among those with weekly headache, 19.6% had never sought medical advice for it. One in six (16.5%: more than two thirds of the 24.4%) had never done so despite agreeing or strongly agreeing that their headache was a burden in their everyday lives (Table 5). Healthcare utilization rate followed headache frequency: the lower the frequency, the higher the proportion who had never consulted (Table 3). The most common reason for not doing so, reported by more than one third (36.8%), was ‘I do not know if my doctor can help me with my headache’ (Table 5). Among those reporting that headache was burdensome in their everyday lives, but who had not sought medical advice, the same reason was given by almost half (45.4%) (Table 6).

**Table 5:** Characteristics of all participants not seeking medical advice, and reasons for not doing so

Enquiry	Response according to reported headache frequency				
**How often do you have headache (past year)?**	**All participants** *n* = 731 (100%)	**At least once a week** *n* = 369 (50.5%)	**A couple of times a month** *n* = 313 (42.8%)	**A couple of times a year** *n* = 44 (6.9%)	**Less frequently** *n* = 5 (0.7%)
**Age, years**					
Mean ± SD	40.5 ± 12.1	38.6 ± 11.8	42.0 ± 11.7	45.1 ± 14.0	53 ± 7.5
**Proportion, n (%)**					
**Gender**					
Male	158 (21.6%)	76 (20.6%)	64 (20.4%)	15 (34.1%)	3 (60.0%)
Female	573 (78.4%)	293 (79.4%)	249 (79.6%)	29 (65.9%)	2 (40.0%)
**Headache type**					
Tension-type headache	444 (60.7%)	239 (64.8%)	185 (59.1%)	19 (43.2%)	1 (20.0%)
Migraine	237 (32.4%)	115 (31.2%)	106 (33.9%)	15 (34.1%)	1 (20%)
Cluster headache	9 (1.2%)	7 (1.9%)	2 (0.6%)	0 (0%)	0 (0%)
Post-traumatic headache	25 (3.4%)	19 (5.1%)	6 (1.9%)	0 (0%)	0 (0%)
Other headache disorder	59 (8.1%)	33 (8.9%)	22 (7.0%)	4 (9.1%)	0 (0%)
Unknown	232 (31.7%)	118 (32.0%)	94 (30.0%)	17 (38.6%)	3 (60.0%)
**I am able to manage my headache attacks well**					
Strongly agree	55 (7.5%)	12 (3.3%)	27 (8.6%)	13 (29.5%)	3 (60.0%)
Agree	266 (36.4%)	107 (29.0%)	141 (45.0%)	16 (36.4%)	2 (40.0%)
Neither agree nor disagree	264 (36.1%)	153 (41.5%)	99 (31.6%)	12 (27.3%)	0 (0%)
Disagree	129 (17.6%)	86 (23.3%)	42 (13.4%)	1 (2.3%)	0 (12.5%)
Strongly disagree	17 (2.3%)	11 (3.0%)	4 (1.3%)	2 (4.5%)	0 (0%)
**My headache is a burden on my everyday life**					
Strongly agree	128 (17.5%)	91 (24.7%)	35 (11.2%)	2 (4.5%)	0 (0%)
Agree	365 (49.9%)	206 (55.8%)	153 (48.9%)	6 (13.6%)	0 (0%)
Neither agree nor disagree	131 (17.9%)	51 (13.8%)	70 (22.4%)	10 (22.7%)	0 (0%)
Disagree	78 (10.7%)	18 (4.9%)	46 (14.7%)	13 (29.5%)	1 (20.0%)
Strongly disagree	29 (4.0%)	3 (0.8%)	9 (2.9%)	13 (29.5%)	4 (80.0%)
**Reason for not seeking care**					
My headache is not bad enough to go to the doctor	162 (22.2%)	48 (13.0%)	92 (29.4%)	19 (43.2%)	3 (60.0%)
I have considered talking to my doctor but have not been able to do so	161 (22.0%)	92 (24.9%)	60 (19.2%)	9 (20.5%)	0 (0%)
I do not know if my doctor can help me with my headache	269 (36.8%)	162 (43.9%)	98 (31.3%)	8 (18.2%)	1 (20.0%)
I do not need my doctor's help to manage my headache	73 (10.0%)	44 (11.9%)	28 (8.9%)	0 (0%)	1 (20.0%)
Other	66 (9.0%)	23 (6.2%)	35 (11.2%)	8 (18.2%)	0 (0%)

**Table 6:** Characteristics of participants not seeking medical advice despite agreeing or strongly agreeing that headache is a burden on everyday life, and reasons for not doing so

Enquiry	Response according to reported headache frequency				
**How often do you have headache (past year)?**	**All participants** *n* = 493 (100%)	**At least once a week** *n* = 297 (60.2%)	**A couple of times a month** *n* = 188 (38.1%)	**A couple of times a year** *n* = 8 (1.6%)	**Less frequently** *n* = 0 (0%)
**Age, years**					
Mean ± SD	39.0 ± 11.0	38.3 ± 11.5	40.1 ± 10.4	38.3 ± 9.7	N/A
**Proportion, n (%)**					
**Gender**					
Male	94 (19.1%)	59 (19.5%)	34 (18.1%)	2 (25%)	N/A
Female	399 (80.9%)	239 (80.5%)	154 (81.9%)	6 (75%)	N/A
**Headache type**					
Tension-type headache	309 (62.7%)	199 (67.0%)	107 (56.9%)	3 (37.5%)	N/A
Migraine	183 (37.1%)	102 (34.3%)	75 (39.9%)	6 (75.0%)	N/A
Cluster headache	7 (1.4%)	5 (1.7%)	2 (1.1%)	0 (0%)	N/A
Post-traumatic headache	16 (3.2%)	15 (5.1%)	1 (0.5%)	0 (0%)	N/A
Other headache disorder	39 (7.9%)	25 (8.4%)	13 (6.9%)	1 (12.5%)	N/A
Unknown	151 (30.6%)	89 (30.0%)	60 (31.9%)	2 (25.0%)	N/A
**Reason**					
My headache is not bad enough to go to the doctor	51 (10.3%)	20 (6.7%)	30 (16.0%)	1 (12.5%)	N/A
I have considered talking to my doctor but have not been able to do so	133 (27.0%)	81 (27.3%)	48 (25.5%)	4 (50.0%)	N/A
I do not know if my doctor can help me with my headache	224 (45.4%)	143 (48.1%)	79 (42.0%)	2 (25.0%)	N/A
I do not need my doctor's help to manage my headache	58 (11.8%)	41 (13.8%)	17 (9.0%)	0 (0%)	N/A
Other	27 (5.5%)	12 (4.0%)	14 (7.4%)	1 (12.5%)	N/A

Of the three quarters (75.6%) who had sought medical advice, those with more frequent headache were more likely to have done so within the last year (declining from 52.0% for weekly headache to 22.4% for yearly headache) (Table 3). Only one quarter (26.1%) agreed or strongly agreed that they felt better equipped to manage their headache following medical consultation, and fewer than one-third (29.2%) felt better informed about their headache (Table 7).

**Table 7:** Perceived quality of care of participants who had sought medical advice

Enquiry	Response according to reported headache frequency				
**How often do you have headache (past year)?**	**All participants** *n* = 2259 (100%)	**At least once a week** *n* = 1512 (66.9%)	**A couple of times a month** *n* = 659 (29.2%)	**A couple of times a year** *n* = 85 (3.8%)	**Less frequently** *n* = 3 (0.1%)
**Age, years**					
Mean ± SD	41.1 ± 11.5	40.5 ± 11.7	41.7 ± 10.8	45.3 ± 11.9	40.0 ± 13.0
**Proportion, n (%)**					
**Gender**					
Male	310 (13.7%)	204 (13.5%)	83 (12.6%)	22 (25.9%)	1 (33.3%)
Female	1949 (86.3%)	1308 (86.5%)	576 (87.4%)	63 (74.1%)	2 (66.7%)
**Headache type**					
Tension-type headache	1381 (61.1%)	979 (64.7%)	378 (57.4%)	24 (28.2%)	0 (0%)
Migraine	1477 (65.4%)	931 (61.6%)	482 (73.1%)	63 (74.1%)	1 (33.3%)
Cluster headache	94 (4.2%)	62 (4.1%)	23 (3.5%)	7 (8.2%)	2 (66.7%)
Post-traumatic headache	291 (12.9%)	262 (17.3%)	29 (4.4%)	0 (0%)	0 (0%)
Other headache disorder	212 (9.4%)	158 (10.4%)	47 (7.1%)	7 (8.2%)	0 (0%)
Unknown	245 (10.8%)	177 (11.7%)	60 (9.1%)	8 (9.4%)	0 (0%)
**Where is your headache treatment/follow-up currently taking place primarily?**					
Primary care	1781 (78.8%)	1113 (73.6%)	586 (88.9%)	81 (95.3%)	1 (33.3%)
Neurologist	176 (7.8%)	138 (9.1%)	35 (5.3%)	2 (2.4%)	1 (33.3%)
Hospital-based services	302 (13.4%)	261 (17.3%)	38 (5.8%)	2 (2.4%)	1 (33.3%)
**Time since last visit**					
< 1 year	1016 (45.0%)	786 (52.0%)	208 (31.6%)	19 (22.4%)	3 (100%)
1–3 years	574 (25.4%)	355 (23.5%)	197 (29.9%)	22 (25.9%)	0 (0%)
> 3 years	669 (29.6%)	371 (24.5%)	254 (38.5%)	44 (51.8%)	0 (0%)
**After consulting the medical doctor for my headache, I can better handle my headache attacks**					
Strongly agree	112 (5.0%)	49 (3.2%)	55 (8.3%)	7 (8.2%)	1 (33.3%)
Agree	477 (21.1%)	275 (18.2%)	175 (26.6%)	26 (30.6%)	1 (33.3%)
Neither agree nor disagree	803 (35.5%)	538 (35.6%)	231 (35.1%)	34 (40.0%)	0 (0%)
Disagree	524 (23.2%)	390 (25.8%)	124 (18.8%)	10 (11.8%)	0 (0%)
Strongly disagree	158 (15.2%)	260 (17.2%)	74 (11.2%)	8 (9.4%)	1 (33.3%)
**After consulting the medical doctor for my headache, I have a better knowledge of my headache**					
Strongly agree	158 (7.0%)	88 (5.8%)	61 (9.3%)	8 (9.4%)	1 (33.3%)
Agree	502 (22.2%)	329 (21.8%)	150 (22.8%)	22 (25.9%)	1 (33.3%)
Neither agree nor disagree	736 (32.6%)	489 (32.3%)	213 (32.3%)	34 (40.0%)	0 (0%)
Disagree	508 (22.5%)	346 (22.9%)	147 (22.3%)	14 (16.5%)	1 (33.3%)
Strongly disagree	355 (15.7%)	260 (17.2%)	88 (13.4%)	7 (8.2%)	0 (0%)

Of those participants in active care, one fifth (21.2%) were being treated in specialist services (secondary care [7.8%] or tertiary care [13.4%]), with the remainder (78.8%) in primary care (Table 3).

### Healthcare utilization (complementary and alternative medicine)

Two thirds (65.3%) of participants overall, and almost three quarters (72.4%) of those with weekly headache, had tried one or more complementary or alternative therapies outside conventional medical care (Table 3). Those with less frequent headache were less likely to have done so (Table 3).

## Discussion

In this nationwide survey of people with headache in Denmark, we identified a low uptake of care despite a perceived need for care. These findings were in parallel with evidence both of suboptimal medical care and of poor awareness of headache among those affected by it, and despite that the HINDER panel was biased towards those more likely to be interested in, to be concerned about and to consult for headache. Evidence of this bias was provided by the gender divide, which was not as the general population, by the high proportion of participants with greater headache-attributed burden, by diagnoses not in accordance with expected proportions, and by the relatively large proportions treated in secondary and tertiary care. For these reasons, our findings of suboptimal care and poor awareness are likely to be amplified among the general population in Denmark.

The essential finding – that one quarter of participants had not sought headache services despite more than two thirds of these same people reporting headache as an everyday burden – is in line with those of a previous Danish survey [11]. As headache services are widely available in Denmark, these findings contribute to a growing body of evidence demonstrating that it is insufficient merely to make services or treatments available [15, 16]. Other countries in Europe, and the United States, have also reported low healthcare utilization, with lesser disease burden similarly associated with lower utilization, and rates far from 100% even among those with highest burden [11, 13, 14].

The reasons for not seeking care that should be beneficial are certainly multiple, high among them being lack of awareness and apparently suboptimal care. More than one third of those not seeking headache care reported not knowing whether their general practitioner would be able to help them, and, more worrying, of those who had sought care, most did not feel it better equipped them to manage their headache attacks. The relatively frequent use of complementary and alternative therapies reflected this. Inadequacy of care is not merely perceived: it is substantiated by our findings of opioid usage, contrary to international and national guidelines, and of low utilization (and ignorance among those with migraine) of triptans. Low adherence to triptan usage was also identified in the Danish Migraine Population Cohort study [17]. In other countries, diagnostic delays, misdiagnosis and otherwise suboptimal management all negatively impact the quality of clinical care [3], consequences of educational gaps occurring first in medical schools and subsequently in residency programmes, themselves due to a paradoxical lack of priority accorded to headache [5, 18]. Despite being relatively well organized, highly resourced and readily accessible [10], Danish headache services are unlikely to be, and evidently are not, entirely free of these deficiencies contributing to lowered expectations and discouraging utilization of headache services.

There are almost certainly other deterrents to care-seeking. Trivialization of headache disorders has been widely reported, and sometimes stigmatization of those who report it. Evidence of these lies in the reports of high rates of work-presenteeism, coupled with lack of support from employers and others [11, 16, 19–23].

### Limitations

The sampling method imposed biases. Not all adults in Denmark are Facebook users, and the recruitment campaign would not have had equal exposure to all, with older adults (above middle age) more likely to be excluded. Interest bias was certain to have influenced willingness to participate, favouring those worst affected. These biases, however, most probably led to conservative findings and underestimates. We could not relate our findings to detailed demographic characteristics, social factors such as financial situation and educational level, or medical histories, since we did not enquire into these. Furthermore, we recognize that self-reported headache diagnoses are of questionable reliability. However, the essential point is that people with headache appear disinclined to seek readily available healthcare despite, therefore, not being reliably informed of what their headache might be. Our categorization of headache frequency into once or more per week, month or year was considered a necessary simplification, but it limited quantitative estimation of disease burden.

## Conclusions

Our findings are indicative of inadequate delivery of headache care in a country that provides free and universal coverage for all its residents. The implications are twofold. First, it is insufficient merely to make headache care available: public education and increased awareness are necessary to encourage uptake by those who would benefit. Second, although Danish headache services are recognized as among the best, they are not free of important gaps. Educational interventions in both pre- and postgraduate settings are necessary, but a prerequisite for these is a resetting of policy priorities, properly to reflect the very high population ill-health burden of headache.

## Data Availability

The data that support the findings of this study are available from the corresponding author, MA, upon reasonable request.

## Abbreviations

DALY: Disability-adjusted life year
LMIC: Low-to middle-income country
HINDER: Headache in Denmark
YLD: Year lived with disability
